# Modulation of Negative Affect Predicts Acceptance of Music Streaming Services, While Personality Does Not

**DOI:** 10.3389/fpsyg.2021.659062

**Published:** 2021-07-20

**Authors:** Max Hilsdorf, Claudia Bullerjahn

**Affiliations:** Department of Social Sciences and Cultural Studies, Institute of Musicology and Music Education, Justus-Liebig-University Giessen, Giessen, Germany

**Keywords:** music streaming services, personality, affect modulation, emotion regulation, technology acceptance model, negative affect, uses of music, music in everyday life

## Abstract

Music streaming services (MSS) offer their users numerous ways of choosing and implementing their individual approaches to music listening. Personality, uses of music, and the acceptance of MSS can be conceptualized as interdependent. This study investigates whether negative affect modulation strategies explain differences in the acceptance of MSS and integrates findings from previous research into a structural equation model. As for measurements, the Big Five Inventory 2, the Inventory for the Assessment of Activation and Arousal modulation through Music, and adapted scales from previous research on the Technology Acceptance Model were used. A convenience sample of 825 participants (24.3 years; 74% females and 89% students) successfully completed an online questionnaire. In total, 89 percent of the sample reported using MSS regularly. The results show that the tendency to modulate negative affect through music is positively influenced by openness and neuroticism. In turn, the tendency to modulate negative affect through music is shown to increase the perceived usefulness of MSS. However, this study failed to replicate the previous findings that openness increases the attitude toward using and that neuroticism decreases the perceived usefulness. This implies that uses of music are more effective than personality traits at predicting the individual acceptance of MSS. However, personality can be viewed as a predictor for uses of music. The interwovenness of stable and situational factors of music choices is supported. MSS seem to assist their users in coping with negative affect in everyday life, increasing wellbeing. MSS should expand their personalization features to optimize user experience with respect to individual uses of music.

## Introduction

The rise of the peer-to-peer file-sharing platform *Napster* in 1999 threw the music industry into what can be regarded as its most severe crisis thus far. It was only with the establishment of music streaming services (MSS) that the ongoing decline of physical music sales could be compensated for with digital revenue (IFPI, 2015). MP3 download platforms, the former bearer of hope, have failed to reach this goal. This begs the question of what made MSS so popular and successful. On the surface, it appears that the ability to interactively engage with a large music catalog that is accessible from anywhere through mobile Internet connection is an objectively valuable quality. Additionally, the ubiquitous *freemium* business model makes MSS significantly cheaper than physical music media or downloads for most users. However, beyond this surface-level point of view, the value of MSS also lies in features that are perceived and valued differently by their users. Extensive ways to choose, organize, and share music through playlists as well as plenty of personalized content are key features of MSS. Yet, users differ considerably in how highly they value certain features (Hagen, 2016; Brown and Krause, 2020). Thus, it is plausible that the acceptance of MSS depends on their user’s personality and preferred uses of music. This study aims to test this assumption by integrating the concepts of technology acceptance, personality, and uses of music into one model.

### State of Research

The functions of listening to music can be broken down into three distinct dimensions: “social relatedness,” “self-awareness,” and “arousal and mood regulation” (Schäfer et al., 2013). Out of these three, “arousal and mood regulation” is “the most important dimension of music listening closely followed by self-awareness” (ibid., p. 6.) The Inventory for the Assessment of Activation and Arousal modulation through Music (IAAM; von Georgi et al., 2006) is a well-validated instrument (von Georgi, 2013) which measures the dimensions “relaxation,” “cognitive problem solving,” “reduction of negative activation,” “fun stimulation,” and “arousal modulation,” each with 11 items. The functions of music listening have been assessed both regarding specific interactions with music (Greb et al., 2018, 2019) and regarding stable preferences for certain functional uses of music (von Georgi et al., 2006; Chamorro-Premuzic and Furnham, 2007). Greb et al. (2018, 2019) found that the functions of specific interactions with music varied more with situational than with interpersonal differences. However, the IAAM, which measures stable tendencies to use music in a certain way or in certain situations, has been shown to predict music preferences and personality (von Georgi, 2013). Affect modulation is not only a means to manage affective states in everyday life. Rather, it is connected to positive health outcomes, such as the number and strength of infections, the number of consultations, and the subjective state of health (von Georgi et al., 2006). With that in mind, it is not surprising that people with mental disorders engage in affect modulation through music more so than those with no mental disorder (Gebhardt et al., 2014). Intriguingly, people with affective disorders seem to primarily make use of the affect modulation strategies “relaxation,” “cognitive problem solving,” and “reduction of negative activation,” all of which von Georgi (2013) locate in the domain of negative affect (Gebhardt et al., 2014). Relating the IAAM to individuals with mental illnesses may be a niche application; however, modulation of negative affect remains one of the main uses of music in everyday life for almost everyone.

Recently, the relationship between music listening and negative affect has been a subject of great interest. Most prominently, the sad music paradox has received much attention (Sachs et al., 2015; Schubert, 2016; Menninghaus et al., 2017; Eerola et al., 2018). This paradox consists of the observation that some people get enjoyment out of listening to music classified as sad and/or which makes them sad. It has been shown that individual differences in empathy (Huron and Vuoskoski, 2020), absorption (Garrido and Schubert, 2011), openness, and extraversion (Vuoskoski and Eerola, 2011; Ladinig and Schellenberg, 2012) predict whether a person tends to experience this paradoxical reaction. The relationship between liking and felt sadness seems to be fully mediated by the feeling of being moved (Vuoskoski and Eerola, 2017). This implies that induced sadness is actually pleasurable if it is interpreted as a feeling of sweet sorrow built on past experiences (Peltola and Eerola, 2016). Nostalgia is another example of such pleasurable affects related to sadness and sweet sorrow. In fact, people highly prone to nostalgia experience sadness as the most common affect induced by music (Barrett et al., 2010). Further, sad music seems to activate mind-wandering (Taruffi et al., 2017). Any task-unrelated thought (TUT) where the mind strays from the current task can be considered mind-wandering (Steindorf, 2020). However, mind-wandering is not a random event, but a form of internally directed cognition (Dixon et al., 2014) and “strongly related to one’s goals, concerns, and experiences in everyday life” (Fox et al., 2015). Thus, it has obvious similarities to the IAAM construct “cognitive problem solving.” However, “cognitive problem solving” is clearly directed toward solving internal problems through self-reflection. Hence, it can never be a case of TUT. Perhaps, they are both forms of rumination, which has been linked to the seeking out sad music (Garrido and Schubert, 2013).

Personality is “the complexity of psychological systems that contribute to unity and continuity in the individual’s conduct and experience, both as it is expressed and as it is perceived by that individual and others” (Caprara and Cervone, 2000, p. 10). The *Five-Factor Model*, also known as the “Big Five,” the “Five Factor Model,” or the “OCEAN model,” is the dominant model of personality (McCrae, 2009). Its five traits are “openness,” “conscientiousness,” “extraversion,” “agreeableness,” and “neuroticism.” In the context of music, personality has been shown to correlate with music preferences (Rentfrow and Gosling, 2003, 2006; Schäfer and Mehlhorn, 2017; Ruth and Müllensiefen, 2020), affect modulation strategies (von Georgi et al., 2006; von Georgi, 2013), creativity (da Costa et al., 2015), creative achievement in the arts (Harris et al., 2019), music taxonomy preferences (Ferwerda et al., 2019), the experience of flow (Heller et al., 2015), performance anxiety (Bullerjahn et al., 2020), and playing related pain (Gembris et al., 2020).

**Figure 1:**
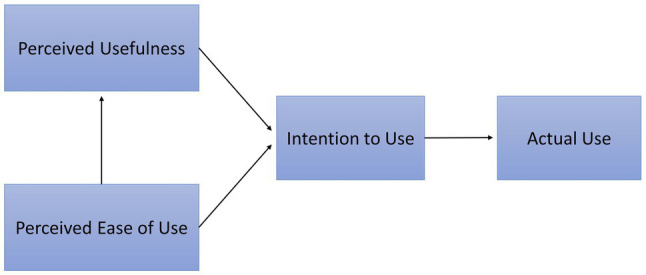
The technology acceptance model (Davis, 1989).

In the context of information systems, technology acceptance is “the repeated decision of a user to use an information system frequently for specific tasks” (Milchrahm, 2002, p. 28). It is typically measured using the *Technology Acceptance Model* (TAM; Davis, 1989), which can be regarded as an adaptation of the *Theory of Reasoned Action* (TRA; Ajzen and Fishbein, 1980). The TAM states that the behavioral intention to use a technology (an antecedent of actual usage) is explained by the perceived usefulness and the perceived ease of use (Figure 1). Meta-analyses have shown that the most common extensions of the TAM are the “subjective norm” and the “attitude toward using” from the TRA, with the latter typically yielding more meaningful results (Schepers and Wetzels, 2007; Avci Yucel and Gulbahar, 2013). The TAM and the TRA as well as their variations have been successfully applied to MSS (Wagner and Hess, 2013; Hampton-Sosa, 2017; Chen et al., 2018; Danckwerts et al., 2018; Chandra et al., 2019; Henning and Ruth, 2020).

“[…] the emergence of MSS that make available millions of tracks to their users, call[s] for intelligent, personalized and context-aware systems to deal with this abundance” (Schedl et al., 2013, p. 535). Thus, in *Music Information Retrieval* (MIR), music recommender systems have been studied extensively in the last decade (Schedl et al., 2013; Soleymani et al., 2015; Airoldi et al., 2016; Prey, 2016, 2019; Eriksson and Johansson, 2017; Pesek et al., 2017). These recommender systems can be distinguished into *system-based* and *user-centric* systems. While the first is concerned with fitting a system to an objective ground truth, the latter is built on the assumption that what matters is the judgments of the users, who do all not subscribe to a universal truth (Schedl et al., 2013). Recently, researchers have observed a shift from system-based to user-centric systems (Schedl et al., 2013; Pesek et al., 2017). Clearly, this shift brings about new ideas and possibilities, but also new challenges. A relevant issue is that – for the lack of an objective ground truth – user-centric systems are difficult to evaluate (Schedl et al., 2013; Strle et al., 2016). Due to a lack of relevant empirical data and competing theories from various disciplines, it is still unclear which attributes and behaviors constitute an adequate user model (Schedl et al., 2013; Strle et al., 2016; Pesek et al., 2017).

A relevant recent study by Ferwerda et al. (2019) touches on most of the previously discussed domains. In study A, they looked into whether personality traits predict the preference for a certain search taxonomy. Study B investigated how an abundance of category choice influences the user experience of MSS and how this relationship is mediated by musical sophistication. Simulating a MSS interface, 297 participants were asked to choose a taxonomy (mood, activity, or genre) to organize the music library by. In an experimental condition, they were randomly offered either 6 or 24 categories within the chosen taxonomy to further specify their request. After the simulation, measures of personality (BFI, study A), musical sophistication (GOLD-MSI, study B), and user experience (adapted from Knijnenburg et al., 2012) were performed. The results of study A show that “openness” predicts a preference for the mood-based taxonomy. Those who decided to filter by activity were high in “conscientiousness” and “neuroticism.” A preference for the genre-based taxonomy was predicted by high “neuroticism.” While the findings regarding “openness” are in line with IAAM research, it is surprising that “neuroticism” did not predict a preference for the mood-based taxonomy, considering that “neuroticism” is characterized by emotional instability and the experience of negative affect. However, the empirical model did not include situational aspects of the decision-making. It is debatable, for instance, whether one’s affective state when answering an online questionnaire sufficiently reflects their affective states in everyday life. Additionally, participants were able to access descriptions of all the taxonomies to inform their decision. The mood description was “Browse for music that fits how you are feeling” (Ferwerda et al., 2019, p. 20163). This clearly implies that this taxonomy aims to help the users maintain their current mood. Considering that this does not constitute a mood modulation, it is not surprising that “neuroticism” did not correlate with this taxonomy. It becomes clear that more research is needed to understand how personality, affective states, and the design of music categorization and recommender systems influence user experience and the acceptance of MSS.

### Research Hypotheses

Hitherto, no studies have investigated the interactions between uses of music, personality, and the acceptance of music listening technologies. However, a considerable amount of research exists on the influence of personality on technology acceptance. It has been shown that neuroticism predicts a lower perceived usefulness of project management tools (Devaraj et al., 2008) and smartphones (Özbek et al., 2014). In their large-scale study (*n* = 1,004) representative for the Norwegian population, Svendsen et al. (2013) did not find this effect for a digital media management tool. Still, “neuroticism” was shown to have a negative effect on other technology acceptance variables related to the perceived usefulness, namely, perceived ease of use and intention to use. Hence, from a theoretical standpoint, these results point in a similar direction as the previously named studies.

*H1*: (N ➔ – PU) “Neuroticism” negatively influences “perceived usefulness of MSS.”

So far, no study has investigated how personality traits relate to the “attitude toward using” in the context of the TAM. However, positive effects on the attitude toward using a free MSS and digital library features were found for “innovativeness” (Wagner and Hess, 2013) and “personal innovativeness in IT” (Wu and Ke, 2015), respectively. These variables are conceptually and empirically related to “openness” (Li et al., 2006).^1^

*H2*: (O ➔ AT) “Openness” positively influences “attitude toward using MSS.”

“Neuroticism” has been consistently found to correlate with affect modulation when not distinguishing between positive and negative affective states (Miranda and Blais-Rochette, 2018). Targeting negative affective states specifically, “relaxation,” “cognitive problem solving,” and “reduction of negative activation” have been repeatedly shown to correlate with “neuroticism” and “openness” (for an overview, see von Georgi, 2013). Because of their high intercorrelations, these three scales can be used as indicators for an overarching construct representing the modulation of negative affect through music (von Georgi et al., 2006, 2009).

*H3*, *H4*: (N, O ➔ MA) “Neuroticism” and “openness” positively influence the “modulation of negative affect through music.”

It can be argued that the perceived ease of use is not an important factor for the acceptance of MSS. MSS are made to be user friendly and should feel intuitive for most people. Empirically, this is reflected in the high mean scores for “perceived ease of use” found in recent studies by Danckwerts et al. (2018) and Henning and Ruth (2020). Regarding the other TAM variables as well as its common extension “attitude toward using,” this study’s hypotheses are in line with previous research.

*H5*: (PU ➔ AT) “Perceived usefulness of MSS” positively influences “attitude toward using MSS.”

*H6*, *H7*: (AT, PU ➔ BI) “Attitude toward using MSS” and “perceived usefulness of MSS” increase “behavioral intention to use MSS.”

So far, no studies have investigated the influence of different uses of music on technology acceptance. Hence, the same applies to the more specific case of the influence of affect modulation strategies on the acceptance of MSS. Users of music technologies differ in their individual needs for these and other features (Hagen, 2016; Brown and Krause, 2020). Thus, the perceived usefulness of MSS must depend on the fit between the affordances and features of a MSS and the individual needs of their potential users. This study argues that MSS are particularly well-suited for people with a high need to modulate their negative affect through music. For this use of music, it is important to be able to access just the right music at just the right time. This is afforded to users by MSS’s large music catalogs and mobile accessibility. In negative affective states, it is also beneficial to be able to choose music without having to make a financial consumer decision, like one would need to for an MP3 download. Furthermore, MSS typically offer hundreds of mood-related playlists as well as the ability for users to create their own. Additionally, personalized music recommendations make it easier for users to find the right music quickly, which may be important in situations of stress or anxiety. Lastly, it is known that affect modulation is a common way of engaging with MSS (Hagen, 2016). Hence, this study hypothesizes:

*H8*: (MA ➔ PU) “Modulation of negative affect through music” increases “perceived usefulness of MSS.”

**Figure 2:**
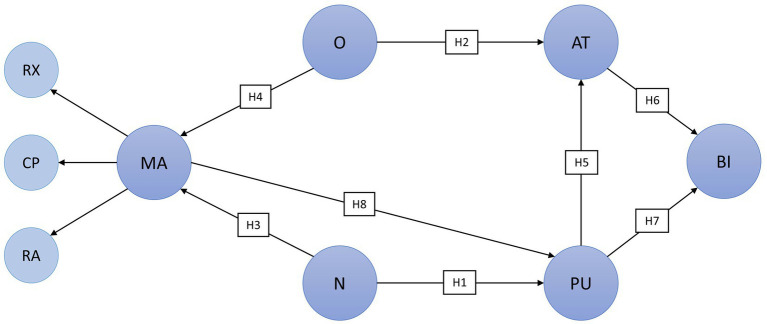
Research model and hypotheses. AT, attitude toward using MSS; BI, intention to use MSS; CP, cognitive problem solving; MA, modulation of negative affect through music; N, neuroticism; O, openness; PU, perceived usefulness of MSS; RA, reduction of negative activation; and RX, relaxation.

The research hypotheses of this study are visualized in Figure 2.

## Materials and Methods

### Materials

All measures for uses of music, personality, and technology acceptance were adapted from prior research. They were measured on a five-point Likert scale. “Openness” (O) and “neuroticism” (N) were measured using the Big-Five Inventory 2 (BFI-2; Soto and John, 2009, translated into German by Danner et al., 2019). This inventory provides 12 items per trait and allows for the measurement of three facets per trait. For O, these are “intellectual curiosity,” “aesthetic sensitivity,” and “creative imagination.” For N, the three facets are “anxiety,” “depression,” and “emotional volatility.” Measurements for the uses of music constructs “relaxation” (RX), “cognitive problem solving” (CP), and “reduction of negative activation” (RA) were taken directly from the IAAM (von Georgi et al., 2009). Each of these scales consists of 11 items.

The *Technology Acceptance Scales* used were not taken directly, but adapted from other studies. In all cases, additional items were added. This allowed for higher reliabilities, a better fit to the peculiarities of MSS, and gave the authors more freedom to exclude non-effective indicators in the structural equation model analysis. The “perceived usefulness” (PU) scale by Wang et al. (2013) was translated into German and expanded from 6 to 11 items. These additional items accounted for the special characteristics of MSS, like exploration, playlists, search options, and recommendations. “Attitude toward using MSS” (AT) and “behavioral intention to use MSS” (BI) were operationalized using adapted versions of the scales used by Dörr et al. (2013). The AT scale was translated into German, and for every existing item, a similar item was constructed. This doubled the number of items from five to ten. Additionally, four items were reversed. The PU scale was translated and expanded in the same way from three to six items, three of which were reversed.

Apart from these core constructs, participants were asked about the MSS they use or have used and whether they use or have used premium subscriptions of MSS. Lastly, the hours of listening to music on a typical day in the week as well as on a typical day on the weekend were assessed on a five-point scale (0–0.5, 0.5–1, 1–2, 2–4, or 4+ hours).

### Participants

A convenience sample of *n* = 997 participated in an online survey. Most of them were acquired through an internal mailing list of Justus-Liebig-University Giessen and through the participants’ networks. A total of 136 participants were acquired through the survey exchange platforms *SurveyCircle* and *Poll-Pool*. Ten *Amazon* gift cards worth 165 € in total were sent to randomly chosen participants after the survey went offline.

All participants who were either speeding (<5th percentile in working time), failed to pass at least one of two *instructional manipulation checks* (similar to Oppenheimer et al., 2009), or did not complete the entire questionnaire were excluded using listwise deletion. This method is acceptable because an increased standard error is tolerable due to the large sample and the missing completely at random assumption is sufficiently met (Urban and Mayerl, 2014, p. 146). In total, 83 percent of all participants met the quality criteria of this study, resulting in a final sample size of 825. Participants were predominately students (89%) and female (74%). The mean age was 24.3 years (*SD* = 6.41, range = [12, 67]). A total of 97 percent of the participants held a higher education certificate (“Abitur”) or a university degree. Ten percent of the sample were or had been in a music related profession or university program. Overall, the sample can be regarded as representative for Justus-Liebig-University Giessen and similar universities (Justus-Liebig-University Giessen, 2020).

### Procedure

The survey was conducted from January 27 to February 13, 2020. On the title page, participants were told that the study was about “the functions of music listening in everyday life” and that it would take approximately 20 min. Additionally, they were informed about their chance to win a gift card if they completed the questionnaire. They were then ensured that their data would be treated safely and anonymously. Lastly, participants were warned that their involvement and concentration would be tested multiple times throughout the survey. Those participants who stated that they had never used a MSS before early in the questionnaire were later asked to answer any questions concerning the perceived usefulness of MSS intuitively. Firstly, the survey required participants to answer all the questions on their socio-demography as well on their quantitative usage of music in general. Subsequently, the core constructs were assessed in the following order: (1) personality (2) uses of music, and (3) technology acceptance. All items within these categories were internally randomized for each participant. All items on the usage of MSS were placed between (1) and (2). Upon completion, participants were able to enter their e-mail address to sign up for the prize game.

The data was analyzed descriptively first to ensure that it meets the quality criteria for structural equation modeling (SEM). Following the three-step procedure proposed by Malone and Lubansky (2012), the data was explored regarding (1) central tendency and variance (2) distributions, and (3) intercorrelations. Data cleaning and descriptive analyses, including all visualizations, were performed in *Python 3.7* using the modules *Pandas 0.2*, *Matplotlib 3.1*, and *Numpy 1.1*. The SEM analyses were conducted in *R 4.0* using the *Lavaan 0.6* package.

## Results

### Descriptive Statistics

#### Music Streaming Usage

Tables 1 and 2 show the descriptive statistics for all variables related to music usage. The hours of listening to music do not differ much between days during the week and on the weekend (see Table 1). Overall, 45 percent of the sample stated no difference between the two. Thirty-five percent listened to more music on the weekend, while only 20 percent stated the opposite. Overall, it appears that music consumption tends to increase on the weekend. However, long listening times are more likely to occur during the week.

**Table 1:** Hours of listening to music during the week and on the weekend.

Hours listening to music	Typical day during the week	Typical day on the weekend
0–0.5	14.67%	12.36%
0.5–1	29.09%	25.58%
1–2	28.24%	30.42%
2–4	18.55%	23.39%
4+	9.45%	8.24%

**Table 2:** Usage of music streaming services.

		Have used before	Use regularly^*^
MSS		94.78%	88.48%
Premium-MSS		75.15%	69.21%
Sum of MSS used	*M*	1.70	1.01
	*SD*	0.902	0.515
*Spotify*		86.91%	74.06%
*Soundcloud*		31.03%	4.97%
*Apple Music*		18.06%	6.42%
*Deezer*		12.85%	1.94%
*Amazon Music*^∗∗^		10.91%	5.58%
*Google Play* or *YouTube Music*^∗∗^		9.22%	7.86%

∗ *At least once per month.*

∗∗ *Not listed in the questionnaire but added by participants through an open question.*

Table 2 reveals that almost the entire sample had prior experience with MSS (95%) and had been using them regularly (89%) at the time the data were collected. In total, 75 percent had used a premium subscription before and 69 percent had been using one regularly. Yet, while participants tended to have used more than one MSS before (*M* = 1.7, *SD* = 0.902), their regular usage typically focused on only one (*M* = 1.01, *SD* = 0.515). *Spotify* was clearly the dominant MSS with a higher percentage of total and regular users than all the other MSS combined (87% > 81%). This difference is even more pronounced with respect to regular usage (74% > 27%). MSS differed in their ability to keep their users engaged. *Soundcloud* had 84 percent fewer regular users in the sample than it had all time users. For other MSS, this decrease is far less pronounced (14.7% for *Spotify* and 14.8% for *Google Play* or *YouTube Music*).

#### Affect Modulation, Personality, and Technology Acceptance

**Figure 3:**
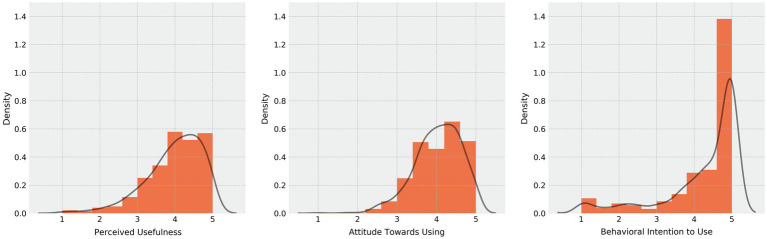
Distributions of the TAM variables “perceived usefulness,” “attitude toward using,” and “behavioral intention to use.” Lines show kernel density estimations.

Table 3 displays the mean, standard deviation, and Cronbach’s alpha, as well as the skewness and kurtosis for each of the scales used. For all scales, high to very high internal consistencies were found (0.825 < *α* < 0.909, *M* = 0.883). The scales regarding uses of music and personality were approximately normally distributed. However, all three technology acceptance scales were significantly leptokurtic and left-skewed. Still, as shown in Figure 3, a graphical representation with kernel density estimation implies that the underlying distribution might be approximately normally distributed but limited by the upper bound of the five-point scale, resulting in a ceiling effect.

**Table 3:** Descriptive scale statistics.

Construct	Scale		# Items	*M*	*SD*	*α*	Skewness	Kurtosis
Uses of music	Relaxation	RX	11	3.37	0.820	0.883	−0.377	−0.023
	Cognitive problem solving	CP	11	3.19	0.881	0.897	−0.166	−0.474
	Reduction of negative activation	RA	11	2.93	0.861	0.885	0.105	−0.340
Personality	Openness	O	12	2.76	0.658	0.883	−0.271	−0.425
	Neuroticism	N	12	3.78	0.569	0.825	0.280	−0.015
Technology acceptance	Perceived usefulness	PU	10	3.98	0.746	0.909	1.200	−1.030
	Attitude toward using	AT	11	4.03	0.594	0.874	0.857	−0.720
	Behavioral intention	BI	6	4.25	1.050	0.906	2.050	−1.679

The correlation analysis included the control variables age (AGE), education (EDU), sex (SEX), all time premium MSS usage (PRE), and the hours of music consumption on a typical weekday (MUS) alongside the scales featured in Table 3. Because of the non-normality of the technology acceptance scales, the non-parametrical *Spearman’s Rho* correlation coefficient was used. Additionally, this method allows for the computation of correlations between metrical, ordinal, and binary data, which were all present in this study (Kubinger et al., 2011, p. 364f.). To transform SEX into a binary variable, four participants who stated “other” as their sex were excluded for the correlation analysis only.

### Correlations

The results of the correlation analysis can be found in Table 4. Because of this study’s large sample size, most correlations were significant at *α* = 0.05. Most of the research hypotheses were already reflected on a correlational level. Only H4 and H5 were not supported by the correlational data. Looking at intra-scale correlations, the items in the uses of music and technology acceptance scales were moderately to highly intercorrelated (0.507 < *r* < 0.633 for the technology acceptance scales, 0.724 < *r* < 0.752 for the uses of music scales). Regarding potential confounding variables, the correlation analysis showed that MSS premium had its highest significant correlations with the technology acceptance scales. However, it is to be expected that those who decide to purchase a MSS premium subscription are fond of the technology. Additionally, buying such a subscription without an intention to use the MSS is irrational. Thus, this correlation seems trivial and irrelevant to the research model. Instead, the time listening to music on a typical weekday was identified as a potential confounding variable for the relationship between the uses of music scales and the technology acceptance scales, because it was significantly and positively correlated with both.

**Table 4:** Correlation matrix.

	O	N	RX	CP	RA	AT	PU	BI	AGE	EDU	SEX	PRE	MUS
**O**	1	−0.071^∗^	0.201^∗∗∗^	0.093^∗∗^	0.072^∗^	−0.028	−0.050	−0.007	−0.036	−0.063	−0.006	−0.030	0.104^∗∗^
**N**		1	0.213^∗∗∗^	0.273^∗∗∗^	0.290^∗∗∗^	0.030	0.025	0.027	−0.110^∗∗^	−0.048	0.181^∗∗∗^	0.040	0.057
**RX**			1	0.747^∗∗∗^	0.724^∗∗∗^	0.163^∗∗∗^	0.235^∗∗∗^	0.144^∗∗∗^	−0.139^∗∗∗^	−0.101^∗∗^	0.136^∗∗∗^	0.109^∗∗^	0.343^∗∗∗^
**CP**				1	0.752^∗∗∗^	0.181^∗∗∗^	0.209^∗∗∗^	0.188^∗∗∗^	−0.185^∗∗∗^	−0.135^∗∗∗^	0.147^∗∗∗^	0.143^∗∗∗^	0.310^∗∗∗^
**RA**					1	0.176^∗∗∗^	0.198^∗∗∗^	0.159^∗∗∗^	−0.161^∗∗∗^	−0.108^∗∗^	0.087^∗^	0.140^∗∗∗^	0.342^∗∗∗^
**AT**						1	0.633^∗∗∗^	0.507^∗∗∗^	−0.113^∗∗^	−0.052	0.077^∗^	0.409^∗∗∗^	0.136^∗∗∗^
**PU**							1	0.552^∗∗∗^	−0.178^∗∗∗^	−0.101^∗∗^	0.074^∗^	0.449^∗∗∗^	0.180^∗∗∗^
**BI**								1	−0.185^∗∗∗^	−0.105^∗∗^	0.063	0.544^∗∗∗^	0.151^∗∗∗^
**AGE**									1	0.495^∗∗∗^	−0.171^∗∗∗^	−0.128^∗∗∗^	−0.161^∗∗∗^
**EDU**										1	−0.013	−0.093^∗∗^	−0.124^∗∗∗^
**SEX**											1	−0.018	−0.080^∗^
**PRE**												1	0.189^∗∗∗^
**MUS**													1

*AGE, age; BI, behavioral intention; CP, cognitive problem solving; EDU, education; MUS, hours of listening to music on a typical weekday; N, neuroticism; O, openness; PRE, usage of a premium MSS; PU, perceived usefulness; RA, reduction of negative activation; RX, relaxation; and SEX, sex.*

∗ *p < 0.05;*

∗∗ *p < 0.01;*

∗∗∗ *p < 0.001.*

### Structural Equation Model

For the SEM analysis, this study followed a two-step-strategy (Urban and Mayerl, 2014, p. 30). With this method, a confirmatory factor analysis (CFA) is conducted to ensure that the model fit is acceptable before performing a full SEM analysis. Due to the non-normality of the technology acceptance scales, the robust maximum likelihood (MLR) estimation was chosen over its regular version.

#### Confirmatory Factor Analysis

**Figure 4:**
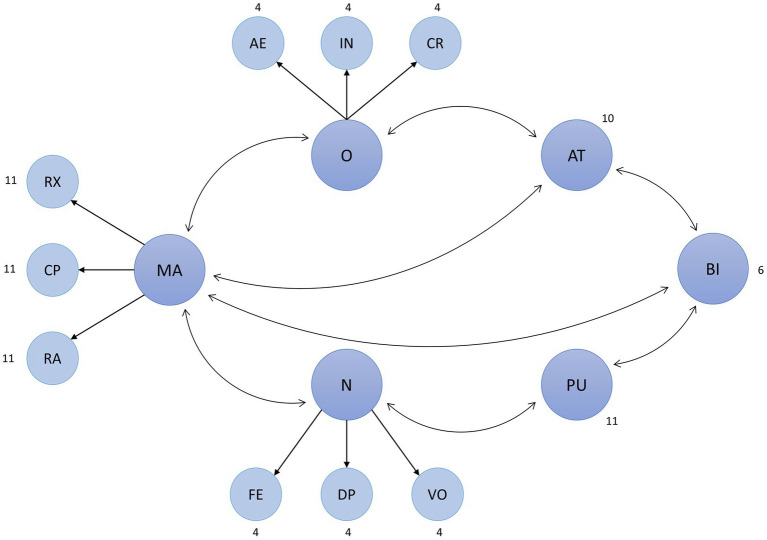
Theory-based CFA model. Resulted in a negative factor variance for the “neuroticism” facet “anxiety.” The numbers next to the latent variables indicate the number of manifest indicator variables for the respective variable. AE, aesthetic interest; AT, attitude toward using MSS; BI, intention to use MSS; CP, cognitive problem solving; CR, creative imagination; DP, depression; FE, fear; IN, intellectual curiosity; MA, modulation of negative affect through music; N, neuroticism; O, openness; PU, perceived usefulness of MSS; RA, reduction of negative activation; RX, relaxation; and VO, emotional volatility.

The first CFA model is displayed in Figure 4. Here, O and N are included in their three-facet structure that the BFI-2 allows for. Furthermore, as laid out in the introduction, RX, CP, and RA serve as indicators for MA. Covariances were added where the research hypotheses would predict such a relationship. All the remaining covariances, including indicator covariances, were assumed to be non-existent.

**Figure 5:**
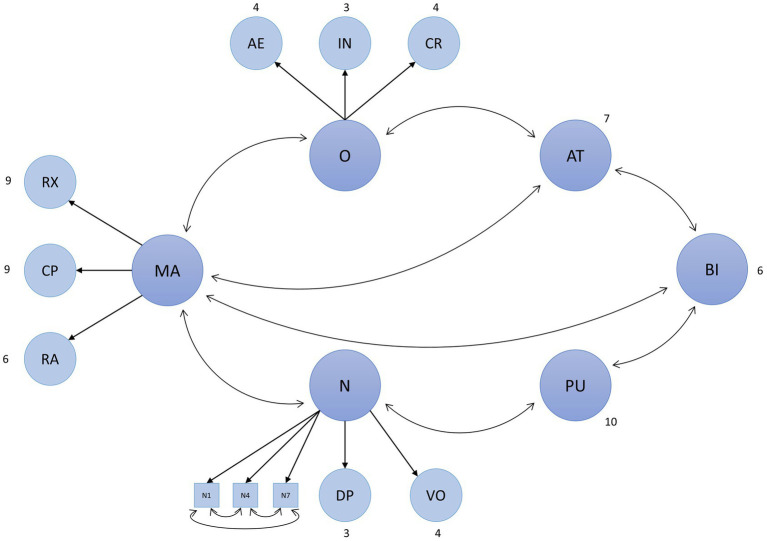
Adjusted CFA model. AE, aesthetic interest; AT, attitude toward using MSS; BI, intention to use MSS; CP, cognitive problem solving; CR, creative imagination; DP, depression; FE, fear; IN, intellectual curiosity; MA, modulation of negative affect through music; N, neuroticism; O, openness; PU, perceived usefulness of MSS; RA, reduction of negative activation; RX, relaxation; and VO, emotional volatility.

The model resulted in a negative variance for the “neuroticism” facet “anxiety” (*s*^2^ = −0.157). This irrational estimation is a common error in SEM analysis known as a “Heywood case.” The low factor loadings of the facet in question (0.508 < *β* < 0.690) may have caused this error (Brown and Moore, 2012, p. 373). The problem was solved by resolving the facet structure for “anxiety,” leaving its former indicators as indicators for “neuroticism” directly. To account for the facet structure in another way, covariances between the four indicators were allowed. The model then converged without any errors ($p_{x^{2}}$ < 0.001, CFI = 0.827, RMSEA = 0.047, SRMR = 0.055). Except for the CFI, all fit measures were already acceptable (West et al., 2012). However, the model still contained numerous low factor loadings. Therefore, to further increase the model fit, 13 indicators with a factor loading lower than 0.55^2^ were removed from the model. The resulting model was significantly improved ($p_{x^{2}}$ < 0.001, CFI = 0.866, RMSEA = 0.045, SRMR = 0.052). Lastly, modification indices with a *χ*^2^ contribution of more than 100 were inspected. Four variables (one from the AT and PU scales, two from the RA scale) showed high factor loadings onto theoretically unrelated latent variables and were thus removed. Additionally, covariances between indicators belonging to the same scale were allowed. This last step, again, significantly improved the model fit ($p_{x^{2}}$ < 0.001, CFI = 0.913, RMSEA = 0.037, SRMR = 0.050). This final model (Figure 5) was accepted for the full SEM analysis.

#### Full Structural Equation Model

**Figure 6:**
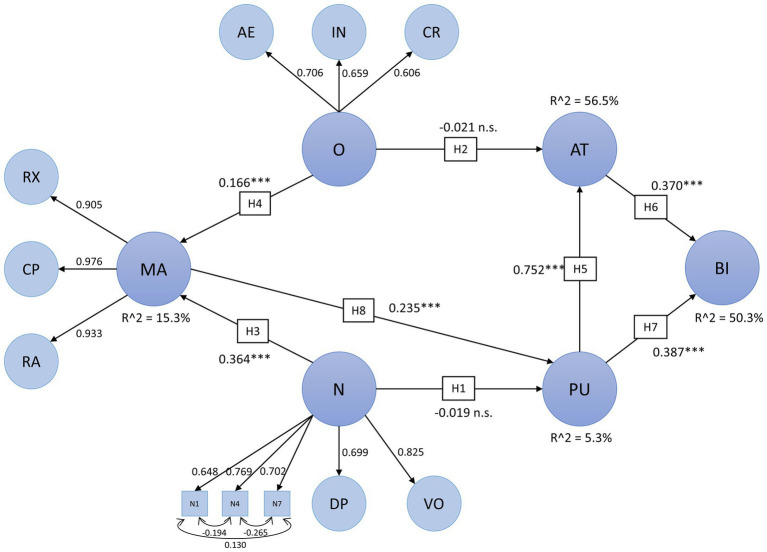
Full SEM results. All path coefficients are standardized regression coefficients. AE, aesthetic interest; AT, attitude toward using MSS; BI, intention to use MSS; CP, cognitive problem solving; CR, creative imagination; DP, depression; FE, fear; IN, intellectual curiosity; MA, modulation of negative affect through music; N, neuroticism; O, openness; PU, perceived usefulness of MSS; RA, reduction of negative activation; RX, relaxation; and VO, emotional volatility. ^∗∗∗^ significant with p < 0.001.

The full SEM results are visualized in Figure 6. Except for H4 and H5, all hypotheses were confirmed by the model. N and O had a moderate (*β* = 0.364, *p* < 0.001) and low (*β* = 0.166, *p* < 0.001) influence on MA, respectively. MA increased PU with a moderate effect (*β* = 0.235, *p* < 0.001). BI was explained moderately by both AT (*β* = 0.370, *p* < 0.001) and PU (*β* = 0.387, *p* < 0.001). PU had a large effect on AT (*β* = 0.752, *p* < 0.001). However, neither O (*β* = −0.021, n. s.) nor N (*β* = −0.019, n. s.) showed significant effects on AT and PU, respectively. Taking mediation into account, multiple indirect effects emerged. Both O (*β* = 0.039) and N (*β* = 0.086) had a minor indirect effect on PU mediated by MA. PU and AT mediated the relationship between MA and BI (*β* = 0.156). Combining direct and indirect effects, PU had a large influence on BI (*β* = 0.665).

To test whether the significant effect of MA on PU was confounded by the amount of time participants listened to music, the hours of listening to music on a typical day in the week were included in the model. It is acceptable to include ordinal data into an ML or MLR estimation if the variable has at least five scale points and is not heavily skewed. Both apply to the variable in question (see Table 1). Listening time had a significant positive effect on both PU (*β* = 0.080, *p* < 0.05) and MA (*β* = 0.328, *p* < 0.001). While this confirms a minor confoundment, the effect of MA on PU only decreased slightly and remained highly significant (*β* = 0.205, *p* < 0.001).

## Discussion

### Discussion of Hypotheses

This study found that the tendency to modulate negative affect through music is a highly significant predictor for the acceptance of MSS. For “openness” and “neuroticism,” which were expected to affect the attitude toward using MSS and the perceived usefulness of MSS, respectively, no such effects were found.

Most findings from previous research were replicated. The tendency to modulate negative affect through music can be operationalized with the factors “relaxation,” “reduction of negative activation,” and “cognitive problem-solving” from the IAAM (von Georgi et al., 2006, 2009). This higher-order construct increases with higher levels of “openness” and “neuroticism” (von Georgi, 2013). An adaptation of the TAM excluding the perceived ease of use and instead including the attitude toward usage was successfully applied to MSS for the first time. With respect to the 50.3 percent of explained variance in the intention to use, this study is similar to previous studies ranging from 36 percent (Henning and Ruth, 2020) to 60 percent (Chen et al., 2018). Furthermore, this model integrates the two existing approaches in research on the acceptance of MSS utilizing TRA (Wagner and Hess, 2013; Chen et al., 2018) and TAM-models (Hampton-Sosa, 2017; Danckwerts et al., 2018; Henning and Ruth, 2020).

The tendency to modulate negative affect through music was shown to have a moderate direct effect on the perceived usefulness of MSS as well as a small indirect effect on the intention to use MSS. Controlling for the time spent listening to music on a typical day, the direct effect decreased slightly but remained highly significant. This supports the idea that the features and affordances of music technologies are perceived differently by users depending on their individual needs (Hagen, 2016; Brown and Krause, 2020). It also strengthens the argument made earlier that MSS are particularly well-suited for affect modulation strategies. Since affect modulation through music is connected to positive health outcomes (von Georgi et al., 2006), MSS may contribute considerably to the success of these strategies and thus to a person’s wellbeing and health maintenance.

Contrary to previous research, personality traits do not seem to influence the acceptance of MSS. Whether on a correlational level or in the SEM, neuroticism and openness were not found to be linked to any of the technology acceptance constructs measured. One explanation for that could be a type-2 error. It is possible that such an error was caused by the ceiling effects observed for all TAM variables (Urban and Mayerl, 2014, p. 17). On the other hand, the personality trait distributions were sufficiently representative of the German population (Danner et al., 2019). Additionally, with this study’s large sample size, it is unlikely that an existing effect would miss statistical significance.

There are also theoretical explanations. Considering that 89 percent of the sample stated regular usage of MSS, it is disputable whether MSS are still perceived as innovations. The hypothesized positive effect of openness on the attitude toward using MSS was based on the assumption that innovativeness as a facet of openness would increase said attitude. If MSS are not perceived as innovations, this effect should vanish. A similar argument might explain why no effect of neuroticism on the perceived usefulness of MSS was found. It is plausible that this hypothesized effect stems from the fear that a new technology may be of insufficient use or lead to decreased productivity on the job. This fear may not exist regarding MSS, because there are hardly any negative consequences to trying out one. Additionally, the advantages and disadvantages of MSS over other music technologies are well known and easily accessible. This may help people high in neuroticism make a decision they feel comfortable with.

### Limitations

Naturally, this study is not without its limitations. To achieve a questionnaire of reasonable length while maintaining high scale quality, single scales were taken out of existing inventories. This means that the effects of any personality trait or affect modulation strategy outside the model were assumed to be non-existent. Hence, it is possible that some existing effects were overlooked. Epistemologically, the data-driven approach of this study’s SEM analysis can be criticized. To achieve a higher theory-data fit, many scale items were excluded. Modification indices were used to allow for the estimation of some covariances. While these modifications are generally acceptable (Urban and Mayerl, 2014), it is possible that a lower model fit should have been accepted to keep the model closer to its underlying theory. The measurement of the TAM variables was flawed, as ceiling effects were found for all of them. It is not clear in which ways those effects influence the results of an SEM analysis. This study’s sample was young (*M* = 24 years). Because the acceptance of MSS negatively correlates with age (see Table 4), acquiring an older sample may have prevented these ceiling effects. Additionally, the young and predominantly female sample acquired in this study is hardly representative for any population except student populations at universities with a focus on stereotypically female fields of study. Even then, the 10 percent of participants who work/study or have worked/studied in a music-related field need to be considered. Lastly, this study makes no theoretical or empirical distinction between free and paid subscriptions to MSS. Because these are different products, the effects found may not apply to both products individually.

### Research Perspectives

This study offers numerous findings relevant to the interdisciplinary discourse between musicology, psychology, and economics. An ongoing debate in music psychology discusses to which extend music preferences are determined by situational aspects or stable preferences. While some researchers highlight situational aspects (Heye and Lamont, 2010; Pagano et al., 2016; Prey, 2019), others view both situational and stable aspects as the building blocks of music preferences (von Georgi, 2013; Hagen, 2016; Greb et al., 2018, 2019). Uses of music as measured in the *IAAM* have both stable and situational components. They reflect stable preferences for certain reactions to situational variables. This study shows that the tendency to modulate negative affect through music can be used to predict music technology preferences. The stable personality traits openness and neuroticism failed to influence technology acceptance directly. However, indirect effects *via* the tendency to modulate negative affect through music were found. Thus, the interwovenness of situational and stable aspects of music preferences is highlighted.

While the relevance of the modulation of negative affect through music is demonstrated, the “how” of the modulation has not been examined. The ways to deal with negative affect through music likely differ from individual to individual, thereby showing how affect modulation is so closely related to stable personality traits. In the context of music listening, *mood management theory* (Zillmann, 1988) states that the logical response to the experience of negative affect would be to find ways to minimize negative affect and maximize positive affect through music in a conditioned response (Knobloch and Zillmann, 2002). “Relaxation” and “reduction of negative activation” from the IAAM clearly point in this direction. However, mood management theory struggles to explain why a significant amount of people choose music to maintain their negative affect (Vorderer and Schramm, 2004). Also, the sad music paradox is incompatible with mood management theory. It is unclear why a person would voluntarily expose themselves to sad music knowing that it will make them feel sad. Yet, it appears that this sadness enables the activation of a feeling of being moved (Vuoskoski and Eerola, 2017) and nostalgia (Barrett et al., 2010), which is perceived as positive. This specific use of music is perhaps connected to the third negative domain of the IAAM, “cognitive problem solving.” That idea is supported by the findings that sad music induces mind-wandering (Taruffi et al., 2017) and that mind-wandering and “cognitive problem solving” could be related as both different manifestations of rumination.

Partly, this study’s results conflict those obtained by Ferwerda et al. (2019). While they did find that openness predicts a preference for the mood-based taxonomy, they found no such effect for neuroticism. The present study would suggest that one who is high in neuroticism would benefit most from a search taxonomy based on mood, as that would best help them deal with situational negative affect. The fact that Ferwerda et al. (2019) contradict not only this study, but also a whole body of IAAM research, leaves two obvious interpretations. Firstly, despite of the IAAM’s proved external validity, it could still be that the IAAM has conceptual flaws. On the other hand, random or conceptual errors in Ferwerda et al.’s (2019) findings may have caused deviations from the established findings. As discussed earlier, Ferwerda et al. (2019) failed to consider the current affective state of their participants. One could argue that since people high in neuroticism experience frequent, but not permanent negative affect, they would choose to answer online questionnaires when little negative affect is present. This is relevant because since participants were recruited through *Amazon Mechanical Turk*, they were free to *not* participate, for example, if they were not feeling well. Then, what would be the benefit in filtering their search by mood, if there is no negative affect to modulate? Of course, the present study’s finding that neuroticism predicts the tendency to modulate negative affect through music can only be situationally measured if negative affect is present. Furthermore, the taxonomies used were not derived from relevant research but drawn from observing how MSS categorize their music. While this is not generally a bad strategy, it is no wonder that this produces discrepancies with established research theories. The mood-based taxonomy, for example, was described as a means to maintain mood, not to modulate it. Strangely, the act of “relaxing” was included in the activity-based taxonomy. In the IAAM, “relaxation” is a dimension of affect modulation. Thus, a replication of Ferwerda et al.’s (2019) study using established classifications and considering affective state as a mediator seems necessary to resolve this problem.

With mobile music listening becoming more and more accessible and widespread, people have developed distinct mobile listening practices (Heye and Lamont, 2010). For example, many listeners use music to create a comfortable and personal space in busy surroundings. This *auditory bubble* has been a subject of interest in recent research (Bull, 2005, 2006; Heye and Lamont, 2010; Hagen, 2016). Because negative affect often occurs in everyday situations (Kalokerinos et al., 2017), it is plausible that people who tend to modulate negative affect through music are also drawn toward creating an auditory bubble to achieve a successful modulation. On the other hand, many ways of modulating negative affect, like sports, drawing, social interaction, or audio-visual entertainment, are not available in some everyday life situations. In searching for an alternative, people may make use of the auditory bubble and thus learn to modulate their affect through music. Hence, the tendency to modulate negative affect through music may cause as well as result from the experience of an auditory bubble.

The high proportion of regular users of MSS (89%) found in this study begs the question whether new methods should be developed to investigate technology acceptance in this domain. This study suffered from ceiling effects for all technology acceptance variables. One means to avoid this problem would be to carefully control for a sample’s age distribution, because MSS acceptance tends to decrease with age. Another solution could be to move from a general acceptance toward targeting specific sub-aspects of the acceptance of MSS. For example, only 69 percent had a paid subscription for a MSS they used regularly. Likewise, the acceptance of certain recommendation features, different kinds of curated playlists, or social features could be investigated separately in the future research.

As for practical implications, the results of this study could help to improve aspects of marketing and personalization of MSS. Firstly, marketing strategies based on personality traits of potential users do not seem promising in the case of MSS. Instead, there lies potential in the analysis of the reasons why users listen to the music they choose. It appears that the fit between the features and affordances of a MSS and a customer’s preferred uses of music partially decides whether a MSS is adopted or not. A deeper level of personalization that responds not only to the choices a user makes, but also to the intentions behind their choices, could improve this fit. User’s preferred uses of music can be either directly assessed or estimated based on their listening behavior. For example, a user who mostly listens to concept albums by a diverse range of bands could be attributed a cognitive listening style. Another user who tends to listen to motivating workout playlists during the day and party playlists at night seems to use music for arousal and affect modulation. Offering these distinct types of listeners a unique and fitting experience is a challenge MSS should attend to.

This leads to the question of how this study can help to improve the ways in which humans and MSS interact. As recommender systems tend to focus more and more on user-centric approaches, the problem of how to model the user has arisen (Schedl et al., 2013; Strle et al., 2016; Pesek et al., 2017). A major implication of this study is that a user model should avoid treating stable preferences and situational factors as two entirely separate domains. Instead, stable tendencies for a certain behavior in a certain situation can be effective predictors of technology acceptance. Situational music preferences must be viewed not only as composed by stable and situational factors, but also as composed by an interaction of both. As this study is partly exploratory, the future research should modify, expand, and test these new ideas. The field of MIR is highly interdisciplinary. For math- or computer science-related fields, it would be worthwhile to implement our findings into recommender system algorithms and to evaluate their effectiveness. Subsequently, MSS need to work on user-friendly ways to obtain the relevant data and to present the new recommendations to their users. For this, research from other fields, like psychology and economics, is needed. It seems clear that if such recommender systems were well-performing, efficient, and user-friendly, this improved user-orientation could be an attractive marketing tool.

## Conclusion

In conclusion, the study implies that uses of music are better predictors of the acceptance of MSS than personality traits. Specifically, the tendency to modulate negative affect through music has a positive direct effect on the perceived usefulness of MSS and a positive indirect effect on the intention to use MSS. This shows that MSS are well-suited for this listening strategy. It was also found that the tendency to modulate negative affect through music is more pronounced for people high in “neuroticism” and “openness.” Both personality traits thus carry an indirect, but no direct positive effect on the acceptance of MSS.

## Data Availability Statement

The original contributions presented in the study are included in the Supplementary Material, further inquiries can be directed to the corresponding author.

## Ethics Statement

The present study was conducted in full accordance with the Ethical Guidelines of the German Association of Psychologists (DGPs) and the German Association of Psychologists (BDP) as well as the Ethical Principles of Psychologists and Code of Conduct of the American Psychological Association (APA). These guidelines suggest that for the type of research reported here, a formal ethics approval is not necessary. The present study only used completely anonymous questionnaires. All e-mail addresses given by participants were stored separately from the questionnaire data. Thus, it was impossible to connect any person to their questionnaire data. Moreover, participants were informed about the aim of the questionnaire, the anonymity of the data, and that participation was voluntary. In accordance with the ethical principles mentioned above, it was not required to obtain written informed consent.

## Author Contributions

MH and CB designed the questionnaire and wrote the manuscript. MH analyzed the data. All authors contributed to the article and approved the submitted version.

### Conflict of Interest

The authors declare that the research was conducted in the absence of any commercial or financial relationships that could be construed as a potential conflict of interest.

## Footnotes

^1^Both variables are conceptually similar. “Personal innovativeness in IT” is described as “the willingness of an individual to try out any new information technology” (Argawal and Prasad, 1998). “Innovativeness” is “the general tendency of consumers to adapt new products” (Wagner and Hess, 2013). Hence, Li et al.’s (2006) findings for “personal innovativeness in IT” can be interpreted as evidence for “innovativeness” as well.

^2^The recommended thresholds of 0.50 or 0.70 (Urban and Mayerl, 2014) would have led to either too few (6) or too many (35) indicators removed. The former would have barely improved the model fit. The latter would have raised concern about scale validities and reliabilities and could be considered as too data driven. Therefore, the lower threshold was raised only slightly to 0.55.
