# Supplementary material for: Modulation of Negative Affect Predicts Acceptance of Music Streaming Services, While Personality Does Not
Source: Front Psychol. 2021 Jul 20;12:659062. doi: 10.3389/fpsyg.2021.659062 (PMC8329084; doi:10.3389/fpsyg.2021.659062)
Supplement: Supplementary file 1 [file Data_Sheet_1.PDF]

Questionnaire Prototype

Hilsdorf & Bullerjahn (2021)

(*GER*)

Hierbei handelt es sich um einen Prototyp des verwendeten Online-Fragebogens. Enthalten sind alle Items des Fragebogens in der korrekten Reihenfolge. Zu bedenken ist, dass die Bereiche „Persönlichkeit“, „Musiknutzung“ und „Akzeptanz von Musikstreaming-Services“ für jede/n Teilnehmende/n intern randomisiert wurden. Der Fragebogen steht nur in deutscher Sprache zur Verfügung. Bei Fragen dient Claudia Bullerjahn als Korrespondentin (claudia.bullerjahn@musik.uni-giessen.de).

(*EN*)

This is a prototype for the online questionnaire used. All items of the questionnaire are included in the correct order. Note that the sections „Persönlichkeit“ (personality), „Musiknutzung“ (use of music) und „Akzeptanz von Musikstreaming-Services“ (acceptance of music streaming services) were internally randomized for every participant. The questionnaire is only available in German. Further questions may be directed towards Claudia Bullerjahn (claudia.bullerjahn@musik.uni-giessen.de).

Persönliche Informationen

1. **Was ist Ihr Geschlecht?** ☐ männlich ☐ weiblich ☐ sonstige
2. **Wie alt sind Sie?** Ich bin ________ Jahre alt.
3. **Was ist Ihr höchster formaler Bildungsabschluss?**
   - ☐ Kein Schulabschluss
   - ☐ Hauptschulabschluss
   - ☐ Realschule (Mittlere Reife)
   - ☐ Gymnasium (Abitur)
   - ☐ Fachhochschulabschluss
   - ☐ Hochschulabschluss (Diplom, Magister, Bachelor, Master)
   - ☐ Promotion
4. **Sind Sie Student*in?** ☐ Ja ☐ Nein
5. **Haben Sie im Bereich Musik gearbeitet oder studiert?**
   - ☐ Ja, das tue ich aktuell.
   - ☐ Ja, und zwar in der Vergangenheit.
   - ☐ Nein, noch nie.

Musikhören

6. **Wie viel Zeit verbringen Sie an einem normalen <u>Wochentag</u> damit, bewusst Musik zu hören?**
   - ☐ 0 - 0,5 Stunden
   - ☐ 0,5 - 1 Stunde
   - ☐ 1 - 2 Stunden
   - ☐ 2 - 4 Stunden
   - ☐ Mehr als 4 Stunden

7. **Wie viel Zeit verbringen Sie an einem normalen <u>Samstag oder Sonntag</u> damit, bewusst Musik zu hören?**
   - ☐ 0 - 0,5 Stunden

- ☐ 0,5 - 1 Stunde
- ☐ 1 - 2 Stunden
- ☐ 2 - 4 Stunden
- ☐ Mehr als 4 Stunden

8. Bitte wählen Sie hier die unterste Antwortoption „mehr als 4 Stunden“ aus.

- ☐ 0 - 0,5 Stunden
- ☐ 0,5 - 1 Stunde
- ☐ 1 - 2 Stunden
- ☐ 2 - 4 Stunden
- ☐ Mehr als 4 Stunden

Persönlichkeit

9. Wie sehr treffen die folgenden Aussagen auf Sie zu?

	Trifft gar nicht zu	Trifft eher nicht zu	Trifft teilweise zu	Trifft eher zu	Trifft voll und ganz zu
Ich bleibe auch in stressigen Situationen gelassen.	☐	☐	☐	☐	☐
Ich bin nicht sonderlich kunstinteressiert.	☐	☐	☐	☐	☐
Ich bleibe auch bei Rückschlägen zuversichtlich.	☐	☐	☐	☐	☐
Ich bin vielseitig interessiert.	☐	☐	☐	☐	☐
Ich kann launisch sein, habe schwankende Stimmungen.	☐	☐	☐	☐	☐
Ich bin erfinderisch, mir fallen raffinierte Lösungen ein.	☐	☐	☐	☐	☐
Ich reagiere leicht angespannt.	☐	☐	☐	☐	☐
Ich kann mich für Kunst, Musik und Literatur begeistern.	☐	☐	☐	☐	☐
Ich bin selbstsicher, mit mir zufrieden.	☐	☐	☐	☐	☐
Ich meide philosophische Diskussionen.	☐	☐	☐	☐	☐
Ich bin ausgeglichen, nicht leicht aus der Ruhe zu bringen.	☐	☐	☐	☐	☐
Ich bin nicht besonders einfallsreich.	☐	☐	☐	☐	☐
Ich mache mir oft Sorgen.	☐	☐	☐	☐	☐
Ich weiß Kunst und Schönheit zu schätzen.	☐	☐	☐	☐	☐
Ich fühle mich oft bedrückt, freudlos.	☐	☐	☐	☐	☐

Es macht mir Spaß, gründlich über komplexe Dinge nachzudenken und sie zu verstehen.	☐	☐	☐	☐	☐
Ich habe meine Gefühle unter Kontrolle, werde selten wütend.	☐	☐	☐	☐	☐
Ich bin nicht sonderlich fantasievoll.	☐	☐	☐	☐	☐
Ich werde selten nervös und unsicher.	☐	☐	☐	☐	☐
Ich finde Gedichte und Theaterstücke langweilig	☐	☐	☐	☐	☐
Ich bin oft deprimiert, niedergeschlagen.	☐	☐	☐	☐	☐
Mich interessieren abstrakte Überlegungen wenig.	☐	☐	☐	☐	☐
Ich reagiere schnell gereizt oder genervt.	☐	☐	☐	☐	☐
Ich bin originell, entwickle neue Ideen	☐	☐	☐	☐	☐

Musikstreaming

10. Welche dieser Musikstreaming-Dienste haben Sie bereits genutzt/ausprobiert?
Mehrere Angaben möglich.

☐ Spotify
☐ Apple Music
☐ Deezer
☐ Soundcloud
☐ Einen andereren, und zwar ____________
☐ Gar keinen Musikstreaming-Dienst

11. Welchen dieser Musikstreaming-Dienste nutzen Sie aktuell regelmäßig (min. ein Mal pro Monat)?
Mehrere Angaben möglich.

☐ Spotify
☐ Apple Music
☐ Deezer
☐ Soundcloud
☐ Einen andereren, und zwar ____________
☐ Gar keinen Musikstreaming-Dienst

12. Nutzen Sie aktuell ein Premium-Angebot eines Musikstreaming-Dienstes?

☐ Ja, und zwar ein kostenpflichtiges Abonnement.
☐ Ja, und zwar ein kostenloses Probe-Abonnement.
☐ Nein, nicht mehr.
☐ Nein, noch nie.

Musiknutzung

13. Wie sehr treffen die folgenden Aussagen auf Sie zu?

**Ich höre Musik, wenn...**	Trifft gar nicht zu	Trifft eher nicht zu	Trifft teilweise zu	Trifft eher zu	Trifft voll und ganz zu
ich mich von allen Lasten befreit fühlen möchte.	☐	☐	☐	☐	☐
ich jemanden vermisse.	☐	☐	☐	☐	☐
ich gereizt bin.	☐	☐	☐	☐	☐
ich vor mich hin träumen möchte.	☐	☐	☐	☐	☐
ich über die Zukunft nachdenken möchte.	☐	☐	☐	☐	☐
ich keinen Ausweg für meine Probleme finde.	☐	☐	☐	☐	☐
ich möchte, dass mir dann alles wieder hoffnungsvoller erscheint.	☐	☐	☐	☐	☐
ich verliebt bin.	☐	☐	☐	☐	☐
ich das Gefühl habe, dass alles nervt.	☐	☐	☐	☐	☐
ich zu mir selbst finden möchte.	☐	☐	☐	☐	☐
ich mich mit jemandem, der mir nahe ist, gestritten habe.	☐	☐	☐	☐	☐
ich meine Gefühle offen äußern möchte.	☐	☐	☐	☐	☐
ich glaube, dass das Leben dann wieder einen Sinn bekommt.	☐	☐	☐	☐	☐
ich mal wieder von mir selbst enttäuscht bin.	☐	☐	☐	☐	☐
ich mal so richtig Dampf ablassen will.	☐	☐	☐	☐	☐
ich alle meine "Wehwehchen" vergessen möchte.	☐	☐	☐	☐	☐
ich nicht mehr weiter weiß und mir alles zu viel wird.	☐	☐	☐	☐	☐
ich aggressiv bin.	☐	☐	☐	☐	☐
ich in meiner eigenen Welt sein möchte.	☐	☐	☐	☐	☐
ich mich von anderen Menschen verletzt fühle.	☐	☐	☐	☐	☐
ich meinen angestauten Ärger richtig raus lassen will.	☐	☐	☐	☐	☐
ich der Realität entfliehen will.	☐	☐	☐	☐	☐

ich über Situationen nachdenken möchte, die mich beschäftigen.	☐	☐	☐	☐	☐
ich nervös bin.	☐	☐	☐	☐	☐
ich meinen Körper bewusst erleben will.	☐	☐	☐	☐	☐
ich versuche, mit meinen Problemen klar zukommen.	☐	☐	☐	☐	☐
ich meine Probleme vergessen will.	☐	☐	☐	☐	☐
ich wieder Kraft bekommen will, damit ich nicht so schnell aufgebe.	☐	☐	☐	☐	☐
ich über mich nachdenken will.	☐	☐	☐	☐	☐
ich nicht mehr weiß, wohin mit meinen Gefühlen.	☐	☐	☐	☐	☐
ich alles ein wenig leichter nehmen will.	☐	☐	☐	☐	☐
ich deprimiert bin.	☐	☐	☐	☐	☐
ich glaube, dass ich im Augenblick andere Menschen verletzen könnte.	☐	☐	☐	☐	☐

Akzeptanz von Musikstreaming-Services

Musikstreaming-Dienste zu nutzen...

14a. ist richtig ☐—☐—☐—☐—☐ ist falsch

14b. ist spannend ☐—☐—☐—☐—☐ ist langweilig

14e. ist schlecht ☐—☐—☐—☐—☐ ist gut

14c. gefällt mir nicht ☐—☐—☐—☐—☐ gefällt mir

14d. ist vorteilhaft ☐—☐—☐—☐—☐ ist nachteilig

14e. ist vernünftig ☐—☐—☐—☐—☐ ist leichtfertig

14f. ist sinnvoll ☐—☐—☐—☐—☐ ist sinnlos

14g. ist unangenehm ☐—☐—☐—☐—☐ ist angenehm

14h. ist aufregend ☐—☐—☐—☐—☐ ist öde

14i. ist dumm ☐—☐—☐—☐—☐ ist klug

15. Wie sehr treffen die folgenden Aussagen auf Sie zu?

	Trifft gar nicht zu	Trifft eher nicht zu	Trifft teilweise zu	Trifft eher zu	Trifft voll und ganz zu
Ich beabsichtige, in den kommenden Monaten einen Musikstreaming-Dienst zu nutzen.	☐	☐	☐	☐	☐

Durch die Nutzung von Musikstreaming-Diensten finde ich schneller die Musik, die ich hören möchte.	☐	☐	☐	☐	☐
Ich gehe davon aus, dass ich in bald einmal Musik streamen werde.	☐	☐	☐	☐	☐
Musikstreaming-Dienste zu nutzen erhöht meine Wertschätzung für Musik.	☐	☐	☐	☐	☐
Es ist unwahrscheinlich, dass ich in den nächsten Monaten einen Musikstreaming-Dienst nutze.	☐	☐	☐	☐	☐
Mit Musikstreaming-Diensten finde ich genau die Musik, die ich gerade brauche.	☐	☐	☐	☐	☐
Ich würde in nächster Zeit ungerne einen Musikstreaming-Dienst nutzen.	☐	☐	☐	☐	☐
Die Nutzung von Musikstreaming-Diensten lässt mich Musik mehr genießen.	☐	☐	☐	☐	☐
Es ist nicht geplant, dass ich in nächster Zeit Musik über einen Streaming-Dienst höre.	☐	☐	☐	☐	☐
Musikstreaming-Dienste bieten mir eine große Vielfalt an geeigneter Musik für mich.	☐	☐	☐	☐	☐
Ich freue mich darauf, in den kommenden Monaten einmal Musik zu streamen.	☐	☐	☐	☐	☐
Insgesamt empfinde ich Musikstreaming als nützlich.	☐	☐	☐	☐	☐
Mit Musikstreaming-Diensten finde ich besser die Musik, die zu mir passt.	☐	☐	☐	☐	☐
Musikstreaming-Dienste sind nützlich, um neue Musik zu entdecken.	☐	☐	☐	☐	☐
Es ist gut, dass man Musik bei Streaming-Diensten bereits sortiert vorfindet (z.B. nach Genre, Stimmung oder Entstehungsjahr).	☐	☐	☐	☐	☐
Musikstreaming-Dienste erlauben mir, Musik sinnvoll zu ordnen und zu organisieren.	☐	☐	☐	☐	☐
Ich kann davon profitieren, dass Musikstreaming-Dienste mir Musik empfehlen.	☐	☐	☐	☐	☐
